# Meroterpenoid Chrodrimanins Are Selective and Potent Blockers of Insect GABA-Gated Chloride Channels

**DOI:** 10.1371/journal.pone.0122629

**Published:** 2015-04-22

**Authors:** Yan Xu, Shogo Furutani, Makoto Ihara, Yun Ling, Xinling Yang, Kenji Kai, Hideo Hayashi, Kazuhiko Matsuda

**Affiliations:** 1 Department of Applied Chemistry, College of Science, China Agricultural University, Beijing 100193, P.R. China; 2 Department of Applied Biological Chemistry, Faculty of Agriculture, Kinki University, 3327–204 Nakamachi, Nara 631–8505, Japan; 3 Graduate School of Life and Environmental Sciences, Osaka Prefecture University, 1–1 Gakuen-cho, Naka-ku, Sakai, Osaka 599–8531, Japan; United States Department of Agriculture, Beltsville Agricultural Research Center, UNITED STATES

## Abstract

Meroterpenoid chrodrimanins, produced from *Talaromyces* sp. YO-2, are known to paralyze silkworm (*Bombyx mori*) larvae, but their target is unknown. We have investigated the actions of chrodrimanin B on ligand-gated ion channels of silkworm larval neurons using patch-clamp electrophysiology. Chrodrimanin B had no effect on membrane currents when tested alone at 1 μM. However, it completely blocked the *γ*-aminobutyric acid (GABA)-induced current and showed less pronounced actions on acetylcholine- and L-glutamate-induced currents, when delivered at 1 μM for 1 min prior to co-application with transmitter GABA. Thus, chrodrimanins were also tested on a wild-type isoform of the *B*. *mori* GABA receptor (GABAR) RDL using two-electrode voltage-clamp electrophysiology. Chrodrimanin B attenuated the peak current amplitude of the GABA response of RDL with an IC_50_ of 1.66 nM. The order of the GABAR-blocking potency of chrodrimanins B > D > A was in accordance with their reported insecticidal potency. Chrodrimanin B had no open channel blocking action when tested at 3 nM on the GABA response of RDL. Co-application with 3 nM chrodrimanin B shifted the GABA concentration response curve to a higher concentration and further increase of chrodrimanin B concentration to10 nM; it reduced maximum current amplitude of the GABA response, pointing to a high-affinity competitive action and a lower affinity non-competitive action. The A282S;T286V double mutation of RDL, which impairs the actions of fipronil, hardly affected the blocking action of chrodrimanin B, indicating a binding site of chrodrimanin B distinct from that of fipronil. Chrodrimanin B showed approximately 1,000-fold lower blocking action on human α1β2γ2 GABAR compared to RDL and thus is a selective blocker of insect GABARs.

## Introduction

**Fig 1:**
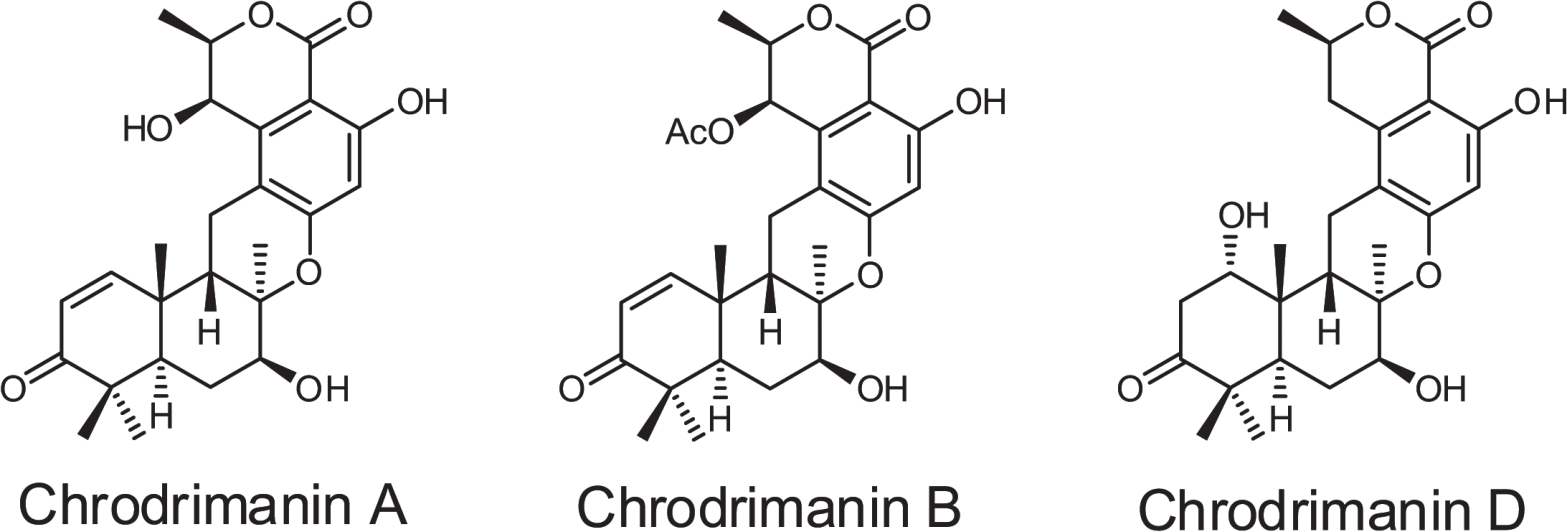
Structures of chrodrimanins A, B and D.

Chrodrimanins are meroterpenoids composed of sesquiterpenoid and polyketide moieties (Fig 1). They were first discovered in 1991 as metabolites of a fungal strain of *Penicillium variabils* and were found to have insecticidal and insect-repelling effects on Lepidoptera [1]. Later, chrodrimanins A–H with their paralyzing actions on silkworm larvae (*Bombyx mori*) were isolated from okara (waste residue from tofu production) that had been fermented with *Talaromyces* sp. YO-2 [2,3]. However, further chemical and mechanistic studies remain to be pursued.

We therefore employed chrodrimanin B (Fig 1), the most insecticidal member of this family, to explore the mode of action in silkworm larvae. Whole-cell patch-clamp electrophysiology has been used to investigate the action of chrodrimanin B on ligand-gated ion channel of larval neurons since rapid paralysis of chrodrimanin-administered larvae pointed to a possible action on ion channels. Given the powerful blocking action on *γ*-aminobutyric acid (GABA)-induced currents, we investigated chrodrimanin actions on a wild-type *B*. *mori* GABAR RDL expressed in *Xenopus laevis* oocytes using voltage-clamp electrophysiology. The RDL-coding gene *rdl* was first isolated from *Drosophila melanogaster* as a gene causal for resistance to dieldrin [4] and can be expressed robustly in *Xenopus* oocytes [5]. Also, the 2′, 6′, and 9′ positions of the second transmembrane region of the RDL GABAR have been shown as important binding sites for noncompetitive antagonists (NCAs) [5,6]. Notably, A2′S;T6′V double mutations in *Drosophila* RDL GABAR confer reduced sensitivity to fipronil, EBOB, picrotoxin, γ-benzene hexachloride (lindane), dieldrin and α-endosulfan [7]. Hence, we also investigated the effects of equivalent A282S;T286V mutations on the blocking action of chrodrimanin B.

We have shown that chrodrimanin B acts as a potent, non-open-channel-blocking antagonist on *B*. *mori* RDL with an IC_50_ of 1.13 nM. It shows competitive actions at low concentrations and non-competitive actions at higher concentrations. We report for the first time that chrodrimanin B exhibited much weaker blocking action on human α1β2γ2 GABAR compared to RDL, and thus may serve as a new lead for the design of safer insecticides.

## Materials and Methods

### Chemicals

Chrodrimanins A, B and D were obtained by purifying the okara fermented by *Talaromyces* sp. YO-2 [2,3]. They were stored as powders at room temperature in Osaka Prefecture University. Their purity was >99%. Chrodrimanins were prepared in DMSO at a concentration of 10 mM and stored at -20°C. They did not decompose in DMSO when stored at this temperature. These stock solutions were diluted with the physiological saline or standard oocyte saline. The final concentration (v/v) of DMSO in the test solutions was 0.1% or lower, which had no adverse effect on the cellular response under investigation. Test solutions of ACh, L-glutamate and GABA were directly dissolved in the bath solutions immediately prior to the experiments.

### Preparation of silkworm larval neurons

The mushroom body neurons of *B*. *mori* larvae were prepared as previously described [8]. The mushroom bodies dissected from the head of silkworm larvae were placed in a Ca^2+^-free medium containing 135 mM NaCl, 3 mM KCl, 4 mM MgCl_2_, 10 mM glucose and 10 mM HEPES (pH 7.3, adjusted with NaOH) supplemented with 50 units ml^-1^ penicillin and 50 μg ml^-1^ streptomycin (Sigma-Aldrich, St. Louis, MO, USA). The excided mushroom bodies were desheathed using a pair of fine forceps. After treatment with 1 mg ml^-1^ Type IA collagenase (Sigma-Aldrich) in the Ca^2+^-free medium, the neurons were dissociated in a culture medium (135 mM NaCl, 3 mM KCl, 4 mM MgCl_2_, 5 mM CaCl_2_, 10 mM glucose, 10 mM trehalose and 10 mM HEPES (pH 7.3) supplemented with 10% fetal bovine serum (Life Technologies, Carlsbad, CA, USA), 50 units ml^-1^ penicillin and 50 μg ml^-1^ streptomycin, followed by incubation in the culture medium on cover slips coated with poly-D-lysine (Sigma-Aldrich) at 25°C for 16–24 h before patch-clamp recording.

### Whole-cell patch-clamp electrophysiology

A patch pipette filled with a solution containing 100 mM KCl, 1 mM CaCl_2_, 4 mM MgCl_2_, 20 mM sodium pyruvate, 10 mM EGTA and 10 mM HEPES (pH 7.3) with a resistance of 5–6 MΩ was used for the experiments [8]. Neurons were superfused continuously at 5 ml min^-1^ with a extracellular buffer solution containing 135 mM NaCl, 3 mM KCl, 5 mM CaCl_2_, 4 mM MgCl_2_, 10 mM glucose and 10 mM HEPES (pH 7.3). The membrane currents were recorded using an Axopatch 200B amplifier (Molecular Devices, Sunnyvale, CA, USA) and were low-pass filtered at 10 kHz using a four-pole Bessel filter and stored on a personal computer using a Digidata 1322A data acquisition system (Molecular Devices). Whole-cell patch-clamp electrophysiology was conducted at 20–23°C at a holding membrane potential of -60 mV. Acetylcholine (ACh), L-glutamate and GABA (Sigma-Aldrich) were applied to the silkworm larval neurons using a U-tube, and chrodrimanin B was applied by either U-tube or bath application.

### Preparation of cDNAs and cRNAs

cDNAs encoding *B*. *mori* GABAR subunit RDL containing exon 3b and exon 6d sequences (Accession number: AB847423) as well as *Homo sapiens* α1 (Accession number: NM_000806.5), β2 (Accession number: NM_021911.2) and γ2 (Accession number: NM_000816.3) were cloned into the pcDNA3.1 vector (Life Technologies). The cDNA encoding the A282S;T286V mutant of the silkworm RDL was prepared by PCR. cRNAs for these cDNAs were transcribed *in vitro* using the mMESSAGE mMACHINE T7 Ultra Transcription Kit (Life Technologies) and dissolved in RNase-free water at a concentration of 1 mg ml^-1^.

### Expression of GABARs in *Xenopus laevis* oocytes

Oocytes dissected from female *Xenopus laevis* were treated with 2.0 mg ml^-1^ Type IA collagenase for 35 min in a Ca^2+^-free standard oocyte saline (Ca^2+^-free SOS) containing 100 mM NaCl, 2 mM KCl, 1 mM MgCl_2_ and 5 mM HEPES 5.0 (pH 7.6), and the follicle cell layer was manually removed using forceps. Then, 23 nl of cRNAs for wild-type silkworm RDL GABAR, the A282S;T286V mutant silkworm RDL GABAR and human GABAR subunits (α1, β2, γ2 = 1:1:1) were injected into oocytes and incubated at 16°C for 1–3 days in SOS containing 100 mM NaCl, 2 mM KCl, 1.8 mM CaCl_2_, 1 mM MgCl_2_ and 5 mM HEPES 5.0 (pH 7.6) supplemented with penicillin (100 units ml^-1^), streptomycin (100 μg ml^-1^), gentamycin (20 μg ml^-1^) and 2.5 mM sodium pyruvate.

### Two-electrode voltage-clamp electrophysiology

*Xenopus* oocytes were continuously superfused with SOS at 18–23°C as previously described [9,10]. Only glass electrodes with a resistance of 1–5 MΩ when filled with 2 M KCl were used for the experiments. Membrane currents were recorded using a GeneClamp 500B amplifier (Molecular Devices) at a holding potential of -80 mV and were stored on a personal computer using a Digidata 1200 data acquisition system (Molecular Devices) and Clampex 8 software (Molecular Devices). Agonists dissolved in SOS were applied to oocytes for 3 s, with an interval of 3 min between applications, to ensure full recovery from desensitization. Chrodrimanins A, B and D were bath-applied to oocytes for 5 min and then co-applied with GABA.

### Analysis of electrophysiological data

The membrane current data was analyzed using Clampfit 9 (Molecular Devices). The concentration-inhibition data for chrodrimanins as well as the concentration-response data for GABA were fitted by non-linear regression analysis using Prism 6 (GraphPad Software, La Jolla, CA, USA) as previously described [10].

## Results

### Effects of chrodrimanin B on silkworm larval neurons

**Fig 2:**
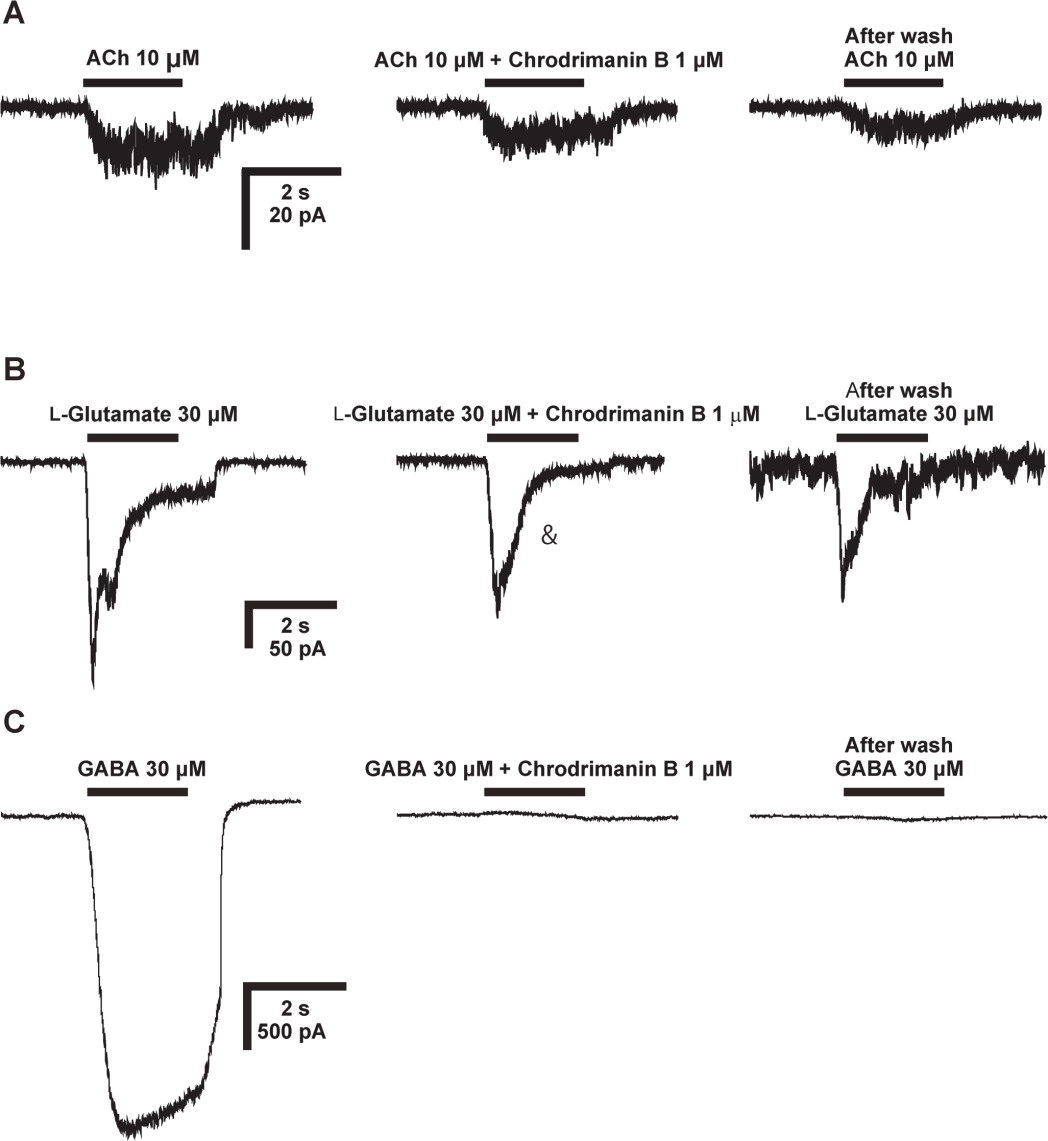
Effects of chrodrimanin B on ACh (A)-, L-glutamate (B)- and GABA (C)-induced currents in the silkworm larval neurons. ACh (10 μM), L-glutamate (30 μM) and GABA (30 μM) were applied for 2 s using a U-tube, whereas chrodrimanin B was bath-applied for 1 min prior to co-application with corresponding agonists. After 3 min wash, each neurotransmitter was challenged again for 2 s.

First, we applied chrodrimanin B alone at 1 μM via a U-tube on the silkworm larval neurons to test for possible agonist actions on ligand-gated ion channels using patch-clamp electrophysiology. Bath-applied chrodrimanin B had no direct effect on the membrane current amplitude. Next, we bath-applied chrodrimanin B at 1 μM for 1 min and then co-applied it with ACh (10 μM), GABA (30 μM) and L-glutamate (30 μM) to test for antagonist actions (n = 3). While chrodrimanin B slightly attenuated the peak amplitude of the ACh- and L-glutamate-induced currents (Fig 2A and 2B), it completely blocked the GABA-induced current (Fig 2C). The blocking action was irreversible (Fig 2C).

### Concentration-dependent blocking actions of chrodrimanins on silkworm RDL GABAR expressed in *Xenopus* oocytes

**Fig 3:**
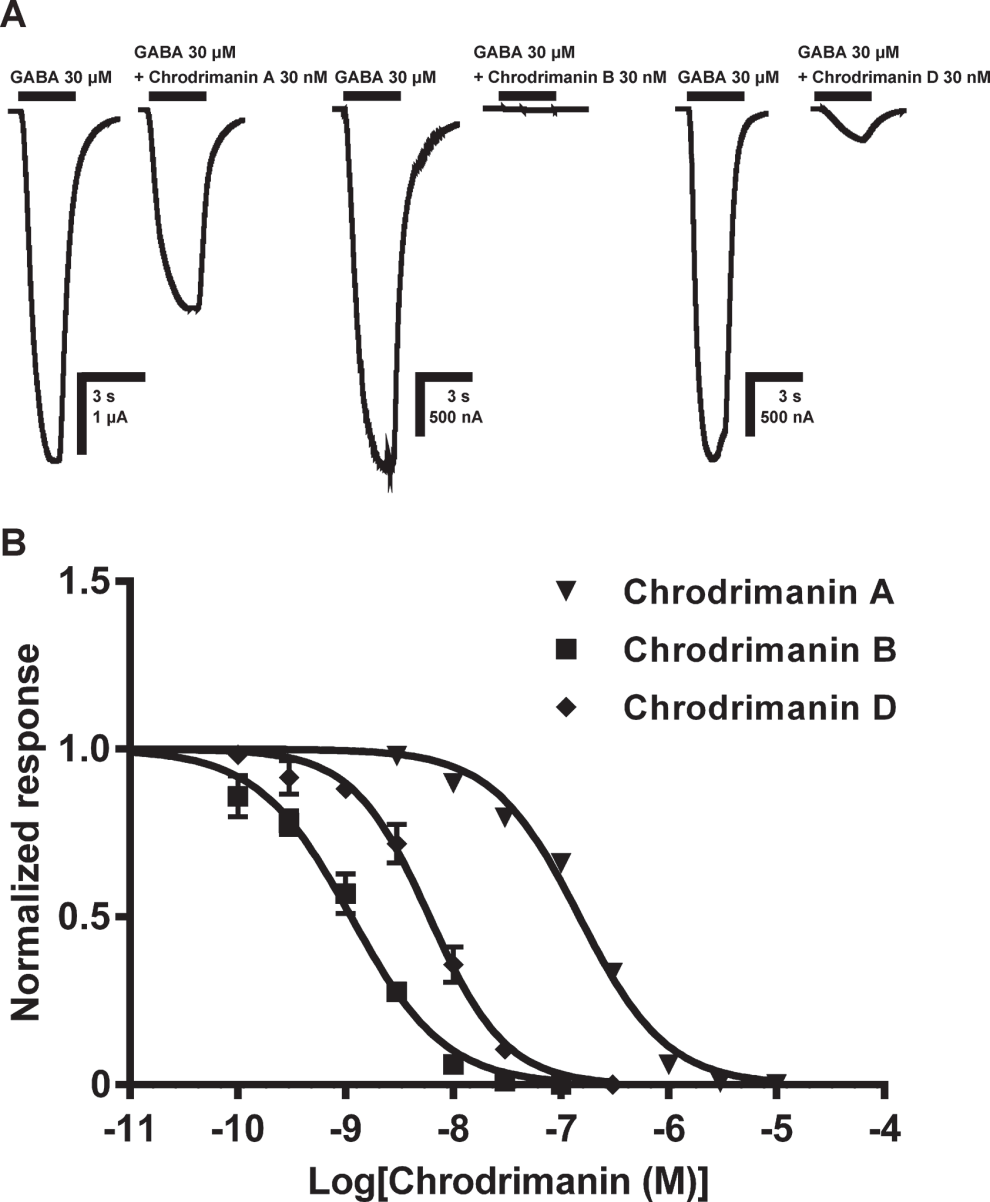
Blocking effects of chrodrimanins A, B and D on silkworm RDL GABAR expressed in *Xenopus* oocytes and concentration-inhibition curves for each compound. (A) After three successive control applications of GABA for 2 s, each chrodrimanin (30 nM) was bath-applied for 5 min and then co-applied with 30 μM GABA to RDL-expressing oocytes for 2 s to record the inward currents in response to GABA. (B) Concentration-normalized response curves for chrodrimanins A, B and D concentration ranges. The peak current amplitude of the response to GABA recorded in the presence of chrodrimanins was normalized to the maximum response to 30 μM GABA recorded before application of chrodrimanins. Each plot represents the mean ± standard error of the mean (SEM) of four experiments. The pIC_50_ (= -log IC_50_) values (mean ± SEM, n = 4) of chrodrimanins A, B and D were 6.83 ± 0.03 (IC_50_ = 148 nM), 8.95 ± 0.04 (IC_50_ = 1.13 nM) and 8.22 ± 0.04 (IC_50_ = 6.01 nM), respectively. The rank order of blocking actions of chrodrimanins was similar to that of their insecticidal actions on the silkworm larvae determined by oral application [2,3].

Having observed an important blocking action of chrodrimanin B on the GABA-induced response of the silkworm larval neurons, we explored further the actions of chrodrimanins A, B and D on the RDL GABAR isoform expressed in *Xenopus* oocytes. We bath-applied each of the chrodrimanins at various concentrations to RDL-expressing oocytes for 5 min and then co-applied each chrodrimanin with 30 μM GABA. When applied at a concentration of 30 nM, chrodrimanins A and D reduced the peak current amplitude of response of GABA by 20% and 89%, respectively, while chrodrimanin B blocked it completely (Fig 3A). Fitting the concentration-blocking activity data by non-linear regression gave IC_50_ values of 148 nM (n = 4, 95% confidence interval (CI) = 130–169 nM), 1.13 nM (n = 4, CI = 0.92–1.38 nM) and 6.01 nM (n = 4, CI = 5.07–7.12 nM) for chrodrimanins A, B and D, respectively (Fig 3B). The rank order of RDL-blocking potency resembled that of their insecticidal activity on silkworm larvae in terms of LD_50_, which was previously determined by oral application using an artificial diet [2, 3].

### Mode of blocking action of chrodrimanin B on silkworm RDL GABAR

**Fig 4:**
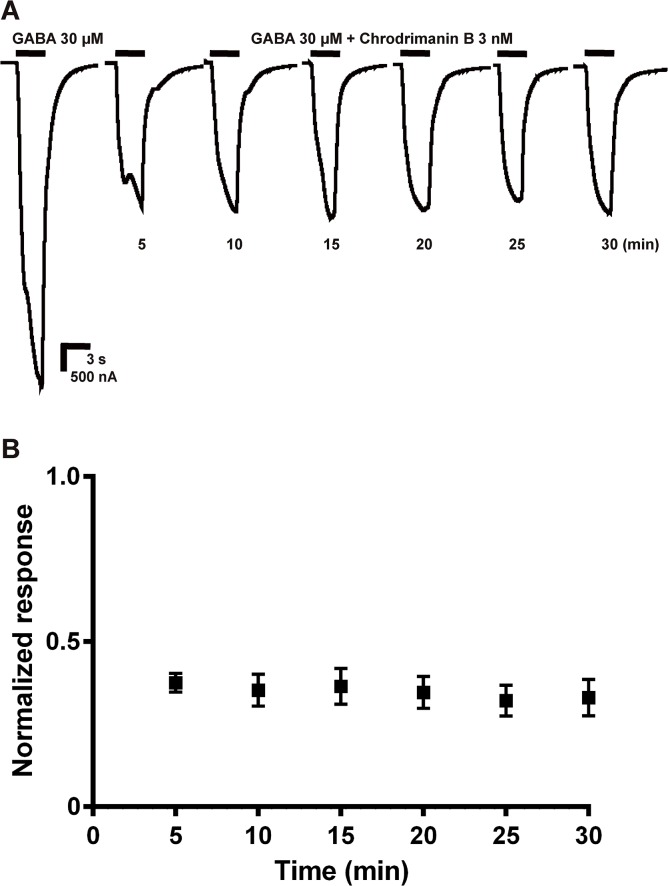
Effects of repeated application of GABA on the blocking action of chrodrimanin B. (A) Traces of the GABA-induced current responses in the presence of 3 nM chrodrimanin B. After three control responses to GABA at 30 μM, chrodrimanin B was continuously bath-applied at 3 nM, during which GABA was also applied at 30 μM every 5 min. (B) Normalized peak current amplitude of GABA responses recorded during continuous application of chrodrimanin B. Each plot represents the mean ± SEM of four separate experiments.

To examine if chrodrimanin B acts as an open-channel blocker similarly to non-competitive antagonists such as dieldrin [11], we continuously applied chrodrimanin B on RDL at a concentration of 3 nM, during which GABA was applied at a concentration of 30 μM every 5 min. The chrodrimanin blocking action was not accelerated by repeated GABA application over a 30 min period (n = 4, Fig 4).

**Fig 5:**
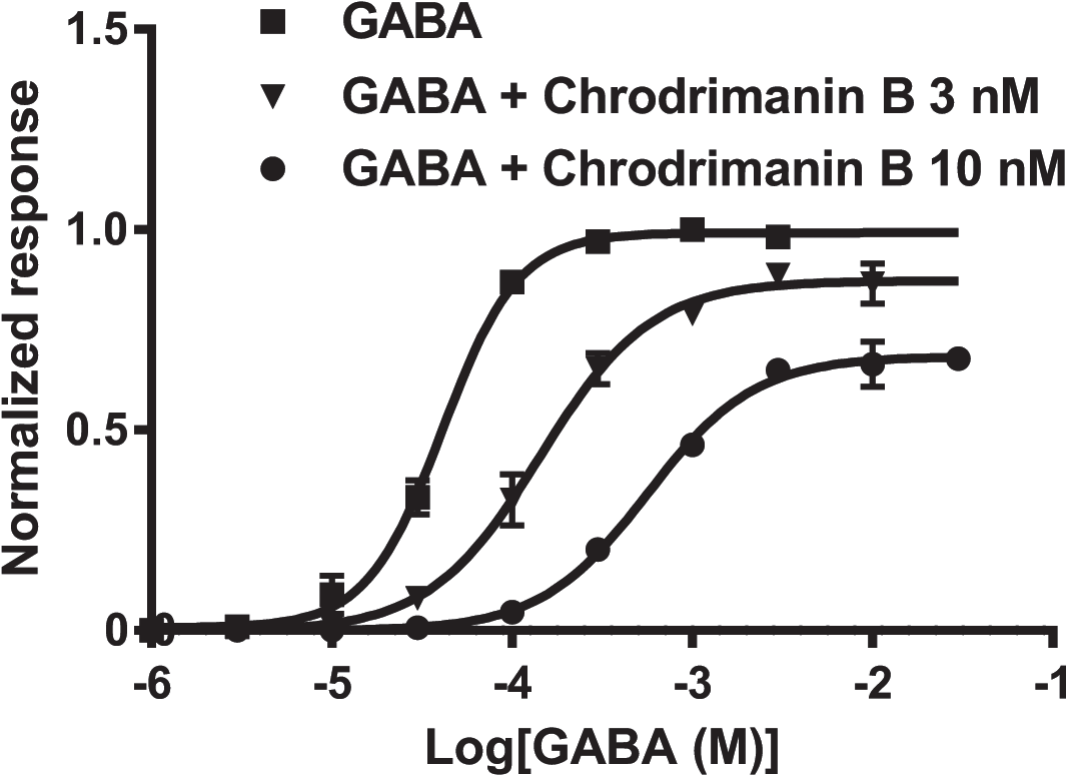
Effects of chrodrimanin B on the concentration-response curve for GABA on silkworm RDL GABAR expressed in *Xenopus* oocytes. The GABA-induced responses were measured at various concentrations in the presence and absence of chrodrimanin B. The EC_50_ value for GABA shifted from 41.1 μM (n = 4, CI = 37.3–45.2 μM) without chrodrimanin B to 143 μM (n = 4, 121–170 μM) with 3 nM chrodrimanin B and to 565 μM (n = 4, 478–668 μM) with 10 nM chrodrimanin B, respectively.

We further investigated the effects of chrodrimanin B at concentrations of 3 and 10 nM on the concentration-response curves for GABA on RDL GABAR expressed in *Xenopus* oocytes. Chrodrimanin B applied at 3 nM significantly shifted pEC_50_ (= -log EC_50_) of GABA from 4.39 ± 0.02 to 3.84 ± 0.04 (n = 4, *p* < 0.05, one-way ANOVA, Dunnett’s test), while it reduced the normalized maximum response to GABA by 12%. Chrodrimanin B applied at 10 nM further shifted the GABA concentration-response curve to the right (pEC_50_ = 3.25 ± 0.04) and reduced the normalized maximum response by approximately 33% (Fig 5).

### Effects of chrodrimanin B and fipronil on A282S;T286V mutant silkworm RDL GABAR

**Fig 6:**
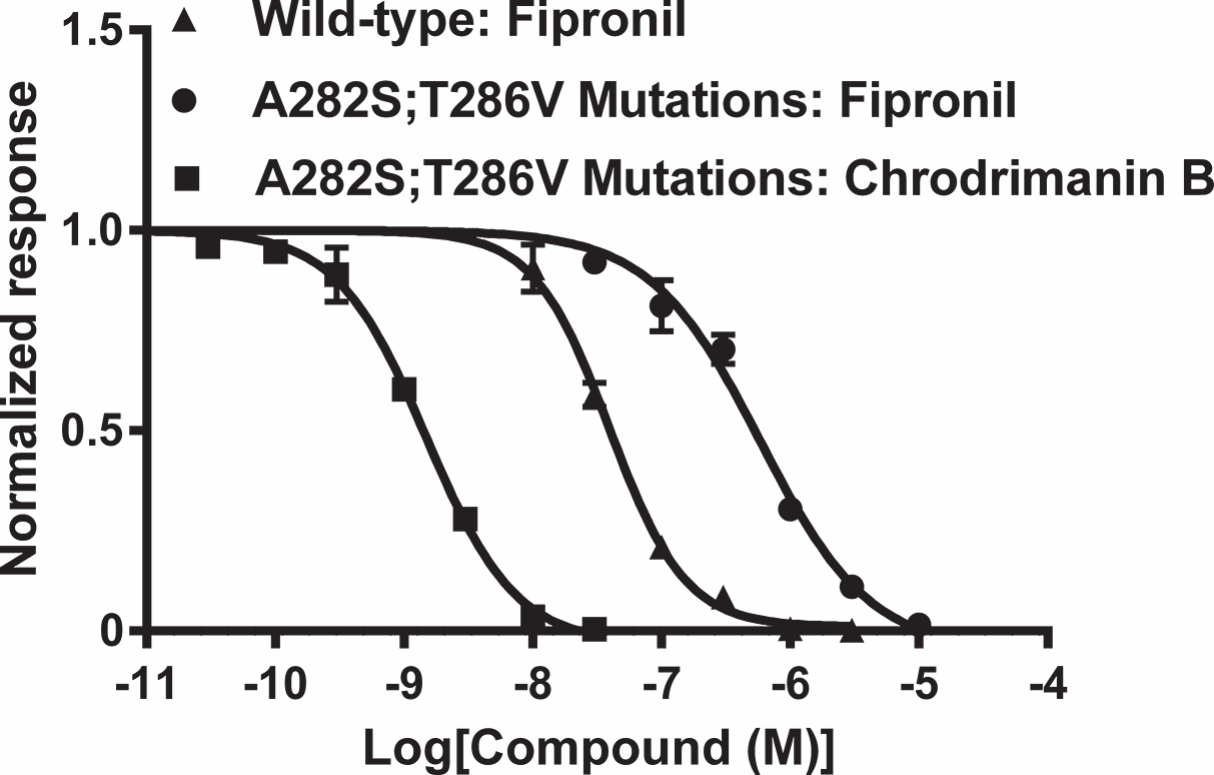
Concentration-inhibition curves for chrodrimanin B and fipronil on A282S;T286V mutant silkworm RDL GABAR expressed in *Xenopus* oocytes. After three control responses to GABA (30 μM for wild-type and 300 μM for A282S;T286V mutant), fipronil was bath-applied for 10 min, during which GABA was also applied every 2 min. The last peak current amplitude recorded in the presence of fipronil was normalized to the maximum response to GABA recorded before application of fipronil. Chrodrimanin B on A282S;T286V mutant was tested using the same method as described for Fig 3. Each plot represents the mean ± SEM of three experiments. The pIC_50_ values (mean ± SEM) of chrodrimanin B on A282S;T286V mutant and fipronil on wild-type and A282S;T286V mutant were 8.83 ± 0.04 (IC_50_ = 1.48 nM), 7.41 ± 0.03 (IC_50_ = 39.4 nM) and 6.24 ± 0.07 (IC_50_ = 583 nM), respectively.

To examine if chrodrimanins target the GABAR chloride channel as in the case for the insecticide fipronil, we investigated the blocking action of chrodrimanin B on a silkworm RDL GABAR with the A282S;T286V double mutation known to reduce the blocking action of fipronil. We bath-applied fipronil to oocytes expressing the mutant RDL for 10 min and then co-applied it with GABA at 300 μM, which is close to the EC_50_ of 303 μM (n = 3, CI = 255–360 μM). Also, we determined the IC_50_ of fipronil for the blocking action on the peak current amplitude of the response to 30 μM GABA of wild-type RDL using a 10-min pre-application protocol. Fipronil blocked the peak current amplitude of the GABA response of the wild-type RDL at an IC_50_ value of 39.4 nM (n = 3, CI = 33.5–46.2 nM), whereas it blocked the mutant at an IC_50_ value of 583 nM (n = 3, CI = 406–837 nM) (Fig 6). Thus, the double mutation reduced the fipronil sensitivity by approximately 14.8-fold. In contrast, chrodrimanin B blocked the peak current amplitude of the response to 300 μM GABA of the A282S;T286V mutant at an IC_50_ value of 1.48 nM (n = 3, CI = 1.21–1.81 nM). No significant difference in IC_50_ of chrodrimanin B was observed between the wild-type and the A282S;T286V mutant. Thus the sites of action of chrodrimanin B and fipronil appear to be distinct.

### Action of chrodrimanin B on human α1β2γ2 GABAR

**Fig 7:**
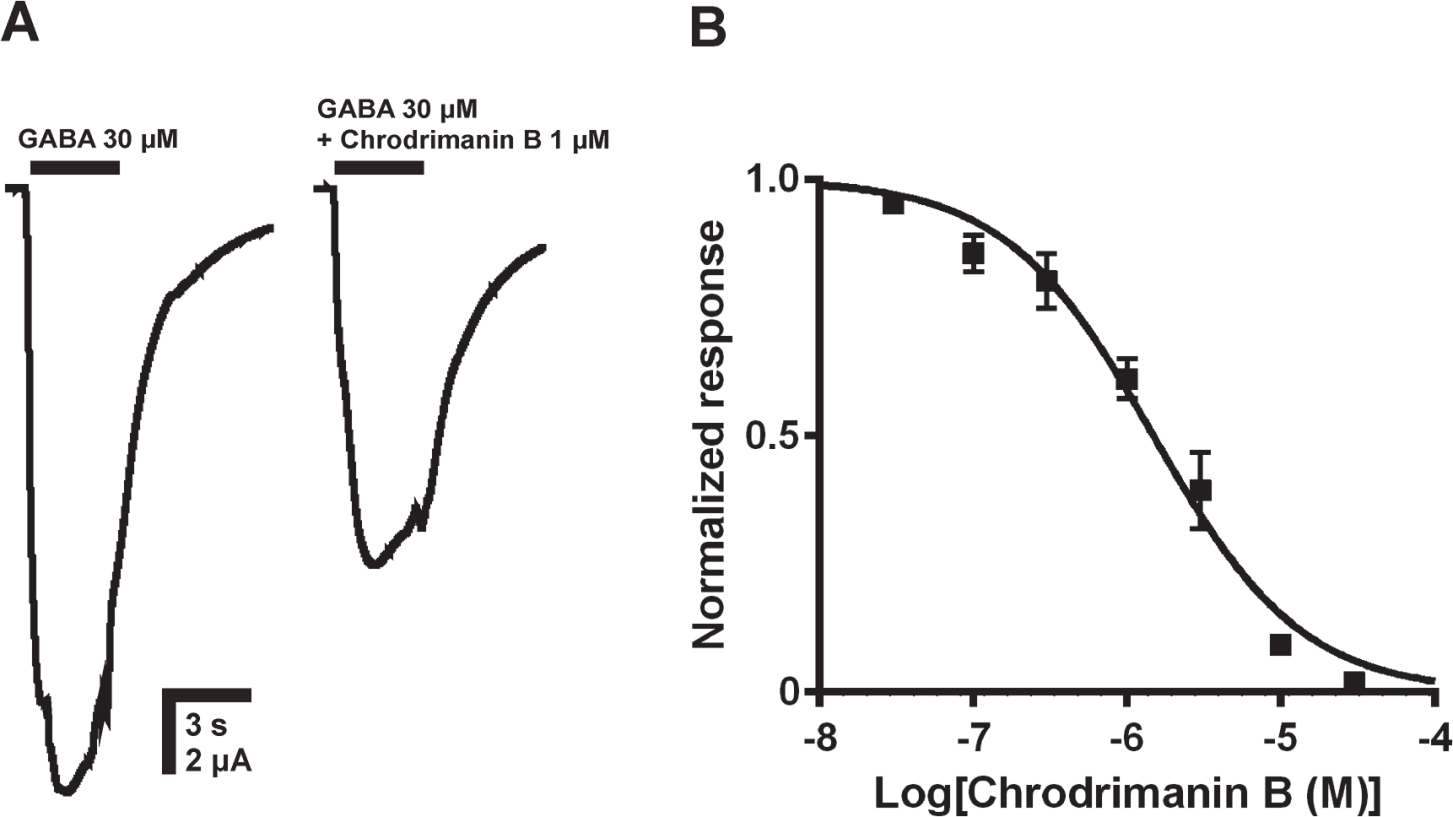
Effects of chrodrimanin B on the GABA-induced responses of human α1β2γ2 GABAR expressed in *Xenopus* oocytes. (A) 1 μM chrodrimanin B was continuously bath-applied for 5 min and then co-applied with 30 μM GABA for 2 s after three successive control applications of GABA. Chrodrimanin B tested at 1 μM blocked the peak current amplitude of the response to GABA of the human GABAR by 38.9 ± 3.9% (n = 3). (B) Chrodrimanin B was bath-applied for 5 min and then co-applied with 30 μM GABA at different concentrations (30 nM—30 μM). Each plot represents the mean ± standard error of the mean of three experiments. The IC_50_ value of chrodrimanin B was 1.48 μM.

To examine if chrodrimanin B is a non-selective GABAR blocker or shows a degree of selectivity for insect GABARs, we also investigated the effects of chrodrimanin B on the human α1β2γ2 GABAR expressed in *Xenopus* oocytes. It was bath-applied at 1 μM for 5 min prior to co-application with GABA at 30 μM, a concentration that was close to the EC_50_ of GABA (38.2 μM, n = 4, CI = 28.2–51.6 μM). Chrodrimanin B reduced the peak current amplitude of the GABA-induced response by only 38.9 ± 3.9% (n = 3) (Fig 7A), which is a much lower blocking action compared to that on RDL. We determined the blocking potency of IC_50_ from the concentration-inhibition curve (Fig 7B) of chrodrimanin B for the human GABAR to be 1.48 μM (n = 3, CI = 1.15–1.91 μM).

## Discussion

Chrodimanins possess five six-membered rings with structural similarities to some other polycyclic natural products such as anisatins [12] and samaderines [13] acting on GABARs. Also the insect-repellent activity of chrodrimanins [1] indicate a possible interaction with the silkworm larval sensilla. Interestingly, a receptor with a GABAR-like pharmacological profile has been reported in insect gustation [14]. However, this is the first report of the actions of chrodrimanins on insect GABARs. Chrodrimanin B exhibited a strong blocking action on the GABA-induced current (Fig 2C), while only slightly affecting the ACh (Fig 2A)- and L-glutamate-induced currents (Fig 2B). This suggests a degree of selectivity for the silkworm larval neuronal GABARs over the nicotinic acetylcholine receptors (nAChRs) and L-glutamate-gated chloride channels (GluCls). In support of such actions, chrodrimanin B strongly blocked the wild-type isoform of RDL GABAR expressed in *Xenopus* oocytes at EC_50_ of 1.13 nM. The rank orders of the blocking potency of chrodrimanin B > D > A was in accord with their toxicity profile to silkworm larvae [2,3], suggesting that the blocking action on the GABAR contributes, at least in part, to insect toxicity. However, discrepancies may be present between the isoforms under investigation in native larval receptor and the isoform expressed in oocytes. Also, we cannot rule out possible actions of chrodrimanins on silkworm histamine-gated chloride channels [15,16,17] as well as pH-sensitive chloride channels [18] expressed in the nervous system.

Since the blocking action was not accelerated by repeated GABA application (Fig 4), chrodrimanin B was not an open-channel blocker, unlike lindane and fipronil. Pre-application for 5 min prior to co-application of 3 nM chrodrimanin B resulted in a shift of EC_50_ for GABA (Fig 5), indicating a competitive interaction with GABA at these concentrations. However, higher concentrations of chrodrimanin B attenuated the maximum GABA response, suggesting that it may also interact with a distinct site from GABA. Similar biphasic blocking actions were observed for the actions of lindane, dieldrin, picrotoxin [19], BIDN [20] and KN244 [21] on RDL and thus chrodrimanin B may partly share its binding site with these NCAs.

To clarify further the mode of action of chrodrimanins, we investigated the effects of A282S;T286V mutation, known to attenuate the chloride-channel-blocking action of non-competitive antagonists, on the RDL blocking action of chrodrimanin B. The A282S;T286V mutation attenuated the blocking action of fipronil, whereas it hardly affected the chrodrimanin action (Fig 6), suggesting that chrodrimanins are unlikely to interact with the GABAR chloride channel in the same way as fipronil. Now that the crystal structure of an invertebrate ligand-gated anion channel in complex with picrotoxinin (PTX) [22], another non-competitive antagonist of GABARs, it will be of interest in future to compare the mode of action of chrodrimanin B with that of PTX as well.

Notably, chrodrimanins resemble bicuculline, a polycyclic GABAR blocker that interacts competitively with mammalian GABARs [23]. SR95531 (gabazine) [24] and 4-substituted 5-(4-piperidyl)-3-isothiazolols [25] have been shown to act as competitive antagonists of insect GABARs. However, their IC_50_s for insect GABARs are higher than 1 μM. If chrodrimanins interact primarily with the orthosteric site, then they are the most potent and selective competitive blocker of insect GABARs. We also examined the selectivity of chrodrimanin B for insect over vertebrate GABARs. Compared to the potent action on RDL, the IC_50_ of chrodrimanin B for the human α1β2γ2 GABAR expressed in *Xenopus* oocytes was about 1,000-fold higher than that for the silkworm RDL. Thus chrodrimanins are novel, candidate insect GABAR-selective blockers. To confirm this, studies on GABARs from insect pest species and other mammalian receptors with other subunit combinations as well as on the other RDL isoforms are needed.

In conclusion, we have shown for the first time that chrodrimainins selectively block GABARs in a different way from fipronil to intoxicate the silkworm larvae, while showing much lower activities on the human α1β2γ2 GABAR as well as on the silkworm larval nAChRs and GluCls expressed in the brain neurons. Chrodrimanins may therefore provide a lead for developing safer pesticides.
